# Drug-Loaded Microbubbles Combined with Ultrasound for Thrombolysis and Malignant Tumor Therapy

**DOI:** 10.1155/2019/6792465

**Published:** 2019-10-01

**Authors:** Qian Gong, Xingxing Gao, Wenfang Liu, Tingting Hong, Chuanpin Chen

**Affiliations:** ^1^Department of Pharmacy, Hunan Cancer Hospital/The Affiliated Cancer Hospital of Xiangya School of Medicine, Central South University, Changsha, Hunan 410013, China; ^2^Xiangya School of Pharmaceutical Sciences, Central South University, Changsha, Hunan 410013, China

## Abstract

Cardiac-cerebral thrombosis and malignant tumor endanger the safety of human life seriously. Traditional chemotherapy drugs have side effects which restrict their applications. Drug-loaded microbubbles can be destroyed by ultrasound irradiation at the focus position and be used for thrombolysis and tumor therapy. Compared with traditional drug treatment, the drug-loaded microbubbles can be excited by ultrasound and release drugs to lesion sites, increasing the local drug concentration and the exposure dose to nonfocal regions, thus reducing the cytotoxicity and side effects of drugs. This article reviews the applications of drug-loaded microbubbles combined with ultrasound for thrombolysis and tumor therapy. We focus on highlighting the advantages of using this new technique for disease treatment and concluding with recommendations for future efforts on the applications of this technology.

## 1. Introduction

Ultrasound is applied to clinical imaging originally, but a series of other biological effects caused by ultrasound can be used in the treatment of solid tumors, leukemia, atherosclerosis, and few other diseases. Ultrasound-based therapy is generally called sonodynamic therapy (SDT), and its mechanism of therapeutic applications is mainly the cavitation effect caused by acoustic waves, that is, mechanical pressure caused by ultrasound, resulting in physical damage to the cytomembrane. Under certain conditions, cavitation directly destroys the cytoskeleton and kills cells, or changes cytomembrane's penetrability to increase drug infiltration [1, 2]. Moreover, ultrasound can act on the sound sensitizer to produce reactive oxygen species (ROS) and kill the cell after the lipid peroxidation [3]. Ultrasound can also induce apoptosis [4], improve antitumor immunity [5], restrain angiogenesis [6], and generate hyperthermia [7]. Conventional surgical treatment or systemic administration commonly leads to series of adverse reactions or irreversible injuries. In contrast to the above conventional treatments, ultrasound can treat the lesion sites in fixed point noninvasively and increase the concentration of drugs in the target tissue, which is the main advantage of ultrasound compared with other treatments in tumor therapy and thrombolysis [8]. Treatment of ultrasound combined with drug-loaded microbubbles, as the hot research topic in recent years, can reduce the uptake of free drugs in nontarget tissues. Meanwhile, ultrasound can destroy microbubbles and release drugs at fixed point and increase the uptake of drugs by cells due to the cavitation effect, thus enhancing the therapeutic efficiency [9, 10].

**Figure 1:**
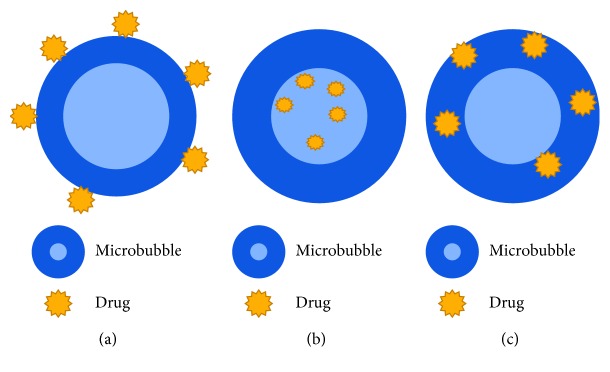
Mechanisms of drug-loaded microbubbles: (a) attached to the surface of the microbubbles; (b) encased inside the microbubbles; (c) embedded in the microbubble membrane.

Microbubbles (MBs) are widely used in the diagnosis and medical treatment of diseases [11–18]. As the main component of ultrasound contrast agent (UCA), microbubbles with diameter less than 10 *μ*m can pass through pulmonary circulation and enhance the contrast of ultrasound imaging in diagnosis. Moreover, microbubbles can improve therapeutic efficiency of focused ultrasound, enhance the heat absorption of tissue, and reduce time required for the ultrasound treatment process [19, 20]. For developing microbubble carriers of drugs, various coating materials including lipids, surface active agents, proteins, and polymers have been used to attach drugs to microbubbles [21–27]. Meanwhile, drugs can be adhered on the surface of the microbubbles, wrapped in the microbubbles, or combined with the membrane by noncovalent bonds (Figure 1) [28]. For instance, hydrophobic drugs such as doxorubicin (DOX), paclitaxel (PTX), and docetaxel can be incorporated into the microbubble shell [29–31]. Specific ligands can be connected to the surface of microbubbles for developing targeting microbubbles [32–35].

Ultrasonic wave can provide a noninvasive, painless, convenient, intuitive, and effective method for medical diagnosis [36–41]. Drug-loaded microbubbles can be destroyed by ultrasound irradiation after reaching the target area so that microbubbles can be busted and can then release drugs. At the same time, ultrasound-induced cavitation can temporarily increase the permeability of the cell membrane, thus increasing the uptake of drugs [42]. Drug delivery with ultrasound relies on the interaction between acoustic wave and biocompatible carrier. Compared with traditional drug treatment, drug-loaded microbubbles in combination with ultrasound can release the drugs in diseased regions, increasing local drug concentration and reducing toxic side effects of drugs [43–46].

**Figure 2:**
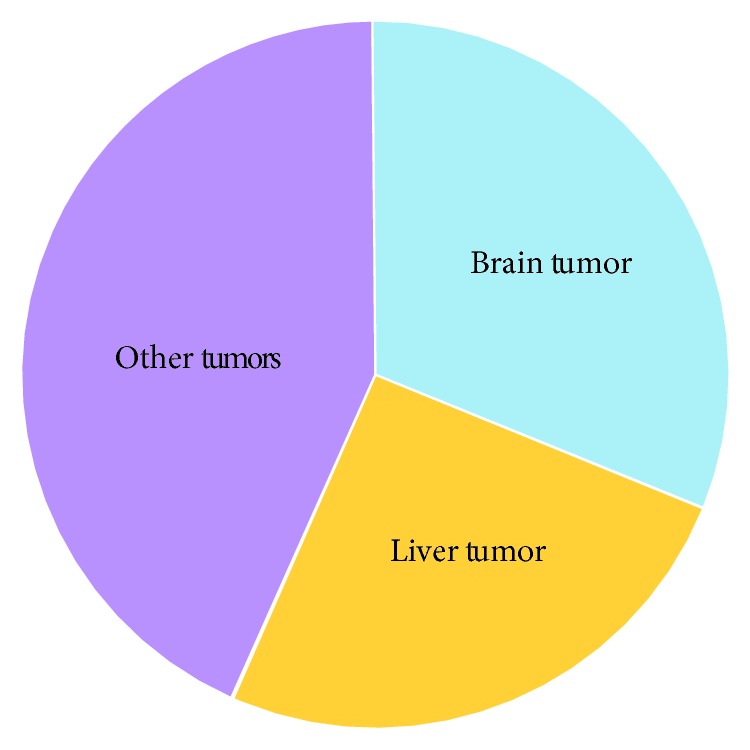
Summary review of the application of microbubbles combined with ultrasound for tumor therapy.

Nowadays, malignant tumor endangers human health seriously [47–50]. Microbubbles combined with ultrasound have recently attracted considerable attention for therapeutic application in tumor treatment.  Figure 2 reviews the application of microbubbles combined with ultrasound for tumor therapy. In this method, drugs carried by microbubbles can reach tumor area via blood circulation and be released in tumor tissue by ultrasound. In comparison with conventional chemotherapy, this strategy presents specific advantages for malignant tumor therapy. Brain tumor is a highly challenging disease for treatment, and mortality from brain tumors have been increasing dramatically. Studies indicate that the combination of drug-loaded microbubbles and ultrasound possess tremendous merits in the treatment of brain tumors. As one of the extraordinarily heterogeneous diseases, liver cancer is becoming a serious medicine issue, and a substantial number of cases are unexplained by risk factors [51–56]. Primary liver cancer ranks the sixth most common cancer and the second leading cause of cancer-related mortality. Among numerous primary liver cancer cases, most cases are hepatocellular carcinoma and intrahepatic cholangiocarcinoma. For liver cancer therapy, preclinical study by using drug-loaded microbubbles combined with ultrasound is important. Since the stroke and acute myocardial infarction caused by thrombosis have resulted in more than half of total global deaths, thrombolytic drugs such as urokinase, streptokinase, and tissue plasminogen activator play an important role in thrombosis treatment [57]. Thrombolytic drugs can activate plasminogen in blood and turn it into active plasmin, inducing the degradation of fibrin and achieving thrombolysis therapy. However, the generated plasmin is not just acting on thrombus, which can cause bleeding and other adverse reactions. Studies suggest that ultrasonic radiation combined with drug-loaded microbubbles can dissolve thrombus directly by releasing drugs at the targeted sites, improving the effect of thrombolysis [58]. Ultrasound-enhanced thrombolysis is a promising strategy for the reperfusion therapies of acute stroke and other thrombus diseases [59, 60].

This article focuses on reviewing the advancements in the treatment of thrombus and tumor by using drug-loaded microbubbles combined with ultrasound. The applications of this state-of-the-art technique for disease treatment are summarized in detail. Moreover, materials used for developing drug-loaded microbubbles will be described. The merits of applying drug-loaded microbubbles combined with ultrasound will be highlighted in this article.

## 2. Drug-Loaded Microbubbles Combined with Ultrasound for Malignant Tumor Treatment

**Figure 3:**
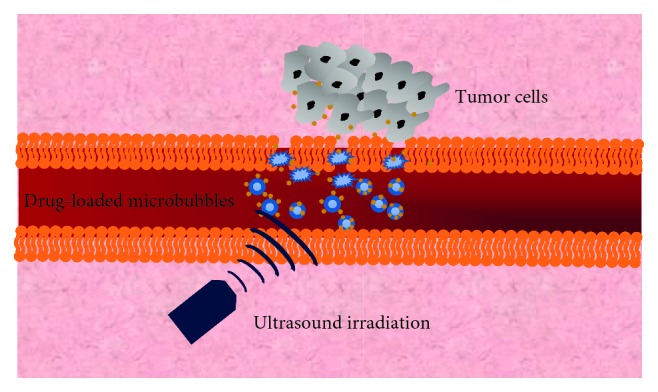
Mechanism of drug-loaded microbubbles combining with ultrasound in the treatment of tumors.

Malignant tumor poses a serious threat to human life and health [61–67]. In the past ten years, there have been tremendous research studies on the combination of drug-loaded microbubbles and ultrasound for drug delivery in animal tumor models (Table 1). Recently, preclinical study of drug-loaded microbubbles combined with ultrasound for tumor therapy has attracted much attention. Large numbers of studies focused on the treatment of brain tumors and liver cancer. In addition, some reports also focused on pancreatic cancer, breast cancer, and other cancers. Compared with the traditional chemotherapy method, chemotherapy drugs carried by microbubbles can reach the tumor area through blood circulation after intravenous injection. After ultrasound irradiation, microbubbles burst in tumor tissue and drugs carried by microbubbles are released into tumor tissue [78], which should be beneficial for the treatment of malignant tumors (Figure 3).

### 2.1. Brain Tumors

Glioma is the most common malignant brain tumor [79–82]. Chemotherapy drugs for the treatment of glioma include carmustine (BCNU), carboplatin, cisplatin, and cyclophosphamide. Despite the increased capillary permeability of brain tumors, traditional methods for the treatment of brain glioma are restricted due to the blood-brain barrier (BBB). BBB decreases the bioavailability of hydrophilic drugs and increases their toxic effect due to the high dose. Ultrasound can instantly open BBB without damage to nerve cells [83, 84]. Microbubbles can be broken by ultrasound, and then, chemotherapy drugs are released and delivered to the tumor sites via crossing the BBB. Escoffre et al. prepared DOX liposome-loaded microbubbles and explored their inhibition ratio to human glioblastoma cells [68]. It was found that ultrasound-triggered release of DOX from the liposome-loaded microbubbles induced a 2-fold decrease of cell survival rate when the peak negative pressure of the acoustic was 200 kPa compared with free DOX or DOX liposome-loaded microbubbles alone. Moreover, the pressure of 400 and 600 kPa could cause 3- and 4-fold decrease of cell survival rate, respectively. The results suggested that microbubbles combined with the ultrasound exhibited synergistic effect on the survival of human glioblastoma cell. Ting et al. prepared BCNU-loaded microbubbles and summarized the way of delivering drug-loaded microbubbles into brain tissue and controlled release triggered by focused ultrasound sonication [69]. The drug was encapsulated in microbubbles so that the circulating half-life was prolonged by 5 times from 16.3 min to 67.5 min. Because the reticuloendothelial system uptake of microbubbles was relatively slow, the drug accumulation in liver decreased by 5 times from 113.57 ± 3.62 *μ*g to 23.87 ± 3.55 *μ*g. Moreover, the treatment efficacy of BCNU-loaded microbubbles combined with focused ultrasound in a rat glioma model was investigated. It revealed that the median survival was extended to 32.5 days and increased about 12% when compared with the control group. Multifunctional microbubbles loaded with DOX and conjugated with superparamagnetic iron oxide nanoparticles were also prepared [70]. The microbubbles could induce the opening of BBB and drug delivery and have been used as dual-imaging contrast agents to determine the drug quantification/deposition. In the referenced study, the vascular endothelial growth factor-targeting drug-loaded microbubbles combined with ultrasound facilitated the opening of BBB, significantly improving the release of targeted drugs [85].

### 2.2. Liver Cancer

Liver cancer is one of the most common malignant tumors nowadays [56, 86–88]. Different types of clinical chemotherapeutic drugs such as mitomycin, 5-fluorouracil, and DOX have been applied for the treatment of hepatocellular carcinoma. The effective rate of chemotherapy drugs on most hepatocellular carcinoma is relatively low. Moreover, many liver cancer patients with hepatic insufficiency are limited to use chemotherapeutic drugs. Drug-loaded microbubbles combined with ultrasound for targeted drug release provides a new approach for the treatment of liver cancer.

Kang et al. investigated the possibility of docetaxel-loaded lipid microbubbles combined with ultrasound to inhibit tumor growth in rabbit liver tumor models [30]. 0.3 MHz nonfocused ultrasound transducer and an intensity of 2 W/cm^2^ was used for the treatment instead of focused ultrasound transducer which was used in most studies. Tumors were exposed to ultrasound irradiation with 10 seconds on followed by 10 seconds off, lasting a total treatment duration of 6 min. The results demonstrated that docetaxel-loaded lipid microbubbles combined with ultrasound could delay tumor cell proliferation, promote apoptosis, and inhibit the growth of VX2 rabbit liver tumor. Taking into account the relatively low drug loading of microbubbles, Li et al. prepared 10-hydroxycamptothecine- (HCPT-)loaded microbubbles, which could display a therapeutic effect at a lower dose compared with other antitumor drugs [71]. In the described work, 1 MHz instead of 0.3 MHz ultrasound transducer was used for the treatment. HCPT, HCPT-loaded microbubbles (HLMs), and HCPT-loaded microbubbles combined with ultrasound (US + HLMs) were applied, respectively, to act on tumor-bearing mice. According to the experimental results, the tumor in the US + HLMs group was significantly smaller, and the tumor inhibition rate was 49.4%, 47.8%, and 70.6%, respectively, compared with the control group. The results showed that drugs could be accumulated in tumor tissue, and the rate of tumor inhibition was significantly increased by using HCPT-loaded microbubbles combined with ultrasound.

In addition to liposomes, polymers like poly(lactic acid) (PLA) were used as drug carrier to prepare DOX-loaded polymer contrast agents [89]. Eisenbrey et al. prepared three types of DOX-loaded PLA-shelled UCA [90]. In this work, drugs were incorporated in the shell or adhered on the surface of agent. It was found that the method of incorporating DOX into the shell was able to achieve desirable particle size distribution, stability, and in vitro enhancement. The size of PLA-shelled contrast agents was reduced to less than 350 nm by using ultrasound. Therefore, the agents could pass through the tumor vascular gap and sustained release drug in tumor stroma. A rabbit liver tumor model was utilized in the in vivo study [72]. The combination of DOX-loaded microbubbles and ultrasound resulted in a decrease of nearly 50% in drug levels in the nontarget region of the liver and an increase of 110% in drug levels within the peripheral of the tumor in vivo studies using the VX2 tumor model. The results indicated that drug delivery and tumor therapy could be achieved by applying DOX-loaded PLA contrast agents combined with ultrasound. However, the drug particle penetration mechanism and sustained release ability was not clear.

### 2.3. Other Tumors

In addition to brain tumors and liver cancer, other cancers, including breast cancer, pancreatic cancer, ovarian cancer, and melanoma have also been investigated with the treatment of drug-loaded microbubbles combined with ultrasound [74, 75, 91–95]. Tinkov et al. prepared DOX-loaded lipid microbubbles and investigated their applicability and efficacy in the treatment of a pancreatic cancer mouse model [31]. Moreover, Ren et al. developed DOX-loaded lipid microbubbles by freeze-drying, and the antitumor effect on human colon adenocarcinoma cell line was explored [73]. The microbubbles prepared by lyophilization were stored in the form of freeze-dried powder, which was convenient for storage. Yan et al. synthesized PTX-liposome-microbubble complexes [74]. In the referenced study, PTX liposomes were coupled to the surface of microbubbles via avidin-biotin linkage, which increased drug-loading capacity of microbubbles. A fluorescent quantum dot was used as model drug to generate liposome-microbubble complexes. And then, the obtained complexes were investigated as ultrasound-mediated drug delivery to treat breast cancer. The results suggested that PTX-liposome-microbubble complexes combined with ultrasound could effectively inhibit the growth of tumor cells. Moreover, DOX-liposome-microbubble complexes joint ultrasound facilitated the delivery of drugs to the sensitive breast cancer cells, preventing multidrug resistance and improving the therapeutic index of the therapy [75].

Concerning that the drug-loaded microbubbles had short cycle times, and the diameter of bubbles was micron level which prevented the bubbles from passing through tumor tissue, Rapoport et al. proposed to develop microbubbles prodrugs at first and then convert it to microbubbles at the tumor site by ultrasound, thus achieving a therapeutic effect [91]. The poly(ethylene oxide)-co-poly(L-lactide) copolymer was utilized to prepare perfluoropentane nanoemulsion which could be converted into microbubbles at a high temperature or ultrasonic irradiation conditions. Subsequently, the human breast cancer, ovarian cancer, and pancreatic cancer cells were transplanted into mice, respectively, to prove the antitumor effect of nanoemulsion combined with ultrasound. However, the tumor recurrence was observed after the first treatment stage, and it was not effective to continue the treatment in the same parts.

Oxygen therapy is commonly used before chemotherapy and radiotherapy. It can promote the oxidation of tumor, improve the drug uptake, and enhance the response of the tumor [96–98]. Microbubbles can be used as a carrier to deliver oxygen and antitumor drugs simultaneously. Wang et al. prepared oxygen and PTX-loaded lipid microbubbles and investigated the efficacy of microbubbles combined with ultrasound in the treatment of ovarian cancer [76, 77]. An in vitro experiment showed that the combination of oxygen and PTX-loaded lipid microbubbles with ultrasound had synergistic effect on hypoxia PTX resistant ovarian cancer cells [93]. It was observed that oxygen and PTX-loaded lipid microbubbles combined with ultrasound could deliver oxygen and antitumor drugs simultaneously, which has a superior antitumor effect [92].

## 3. Drug-Loaded Microbubbles Combined with Ultrasound for Thrombolysis

Cardiac-cerebral thrombosis diseases endanger human health and life safety seriously [99–102]. The stroke and acute myocardial infarction caused by thrombosis have resulted in more than half of total global deaths, which are far more than the deaths caused by cancer, infectious diseases, or respiratory diseases [103]. Thrombolytic drugs applied in clinical treatment such as streptokinase, urokinase, and tissue plasminogen activator (tPA) can achieve thrombolysis therapy by activating plasminogen in blood and turning it into active plasmin. Then fibrin, which is the composition of the thrombus skeleton, can be degraded by the active plasmin. However, since plasminogen flows throughout body blood vessels, plasmin generated by the combination of plasminogen and drugs is not just acting on thrombus, leading to a poor thrombolytic effect and causing bleeding and other adverse reactions [104].

It has been demonstrated that ultrasonic radiation can dissolve thrombus directly or improve the effect of thrombolysis [105–107]. Thrombolytic efficacy can be further improved by microbubbles in combination with ultrasound due to the cavitation nuclei formed in the ultrasound region [108–111]. Microbubble UCA which carries thrombolytic drugs can achieve targeted thrombolysis by using high-intensity ultrasound to break microbubbles and release drugs.

**Figure 4:**
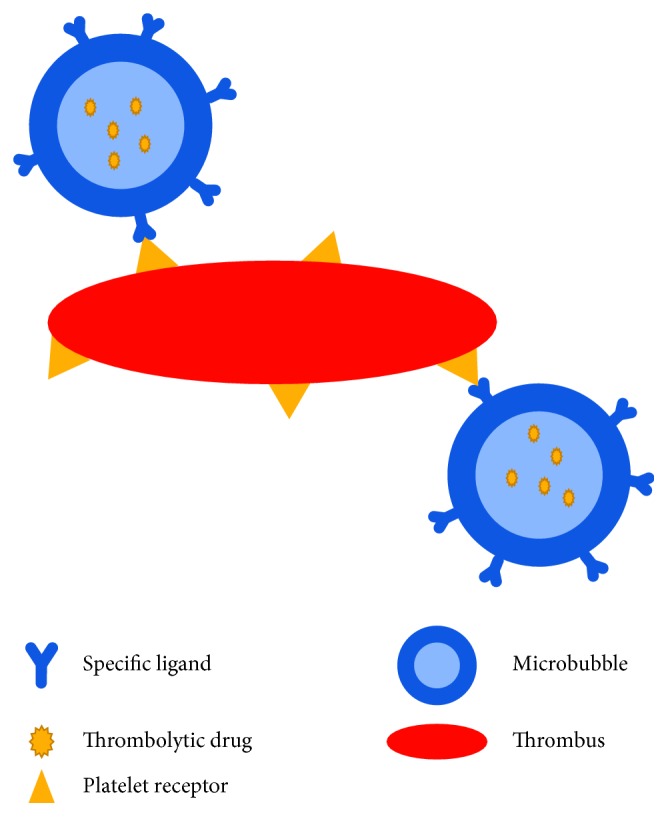
Schematic illustration of thrombus specific targeted microbubbles.

In 2006, Molina et al. suggested that tPA-loaded microbubbles combined with ultrasound could dissolve intravascular thrombus [112]. Particularly, microbubbles with specific ligands on the surface that recognize platelet surface receptors have better therapeutic effect on thrombus (Figure 4). For patients with middle cerebral artery occlusion, they found that tPA-loaded microbubbles combined with ultrasound had better thrombolytic efficiency than tPA/ultrasound or ultrasound alone. The specific ligand Arg-Gly-Asp-Ser (RGDS), which specifically recognizes platelet glycoprotein (GP) IIb/IIIa receptor, can be covalently bound to the surface of microbubbles to develop thrombus specific targeted microbubbles [28]. Therefore, microbubbles can bind with thrombosis specifically and release drugs by ultrasound in local area, achieving the targeted thrombolytic effect. Hua et al. prepared microbubbles loaded with tPA and RGDS using the freeze-drying method [113]. A rabbit femoral artery thrombosis model was utilized to study the thrombolysis effect in vivo. The pulsed ultrasound was emitted with a frequency of 2 MHz, intensity of 1.8 W/cm^2^, pulse repetition frequency of 15 Hz, and duty cycle of 95%. The diagnostic ultrasound was emitted with a frequency of 2 MHz. Compared with using ultrasound alone, targeted drug-loaded microbubbles combined with pulse ultrasound could achieve higher recanalization rate with a low-dose of tPA. The reduction of tPA dose reduced the risk of bleeding. However, the combination of targeted drug-loaded microbubbles with diagnostic ultrasound did not obtain a satisfactory thrombolytic effect. The results suggested that the monitoring and treatment could not be carried out simultaneously [114]. Hagisawa et al. also covalently bound RGDS on the surface of microbubbles so that they could recognize platelet GP IIb/IIIa receptor in thrombus in vivo [115]. It was found that the microbubbles possessed active targeting enrichment capabilities and could improve the imaging contrast. In vivo thrombolysis experiments showed that recanalization rate could reach 90% when combining targeted drug-loaded microbubbles with high-intensity and low-frequency ultrasound (frequency of 27 kHz and intensity of 4.0 W/cm^2^) [116]. Recently, Wang et al. introduced targeted theranostic microbubbles in a rat model of carotid thrombosis [11]. An antibody against the platelet GP IIb/IIIa was developed. Subsequently, the antibody and urokinase plasminogen activator were connected onto microbubbles, and they could recognize thrombus location specifically, achieving real-time monitoring progress of thrombolytic therapy.

## 4. Conclusions

Ultrasound can be used to detect lesions in the tissue, a mathematical model based on doublet mechanics was able to distinguish the difference in cell size and elastic moduli of malignant breast tissue from normal breast tissue [117, 118]. However, when the ultrasound acts on the body tissue, there will be different degrees of attenuation, while the addition of microbubbles can improve the acoustic response to some extent. The combination of targeted drug-loaded microbubbles with ultrasound facilitates drug delivery and tumor targeting, enhancing ultrasound imaging and intracellular drug release. Moreover, ultrasound-mediated microbubble destruction technology is safe and noninvasive. This new technique has shown a desirable therapeutic potential in thrombolysis and tumor therapy.

Most of the related studies on drug-loaded microbubbles focus on how to load the target drugs into microbubbles successfully and on the treatment efficiency of different disease models. There are little systematic discussions on the related factors affecting the therapeutic efficiency of them. From the characterization of the drug-loaded microbubbles preparation, series of physical and chemical properties may be the factors affecting the therapeutic efficacy, such as the size of microbubbles, drug-loading capacity, entrapment efficiency, and microbubble components [119]. Additionally, the drug release of drug-loaded microbubbles based on ultrasound also depends on a range of parameters applied by ultrasound, which closely affects the drug accumulation in target tissue [120].

Ultrasound combined with microbubbles can temporarily open the BBB, thus increasing the release of drugs in brain. At lower peak-rarefactional pressures (PRP), the volume of BBB opening induced by the 6–8 *μ*m microbubbles was greater than that induced by the 4–5 *μ*m microbubbles and smaller microbubble diameters inducing the closing timeline being significantly different than with larger microbubbles. As the PRP increases, the differences in BBB opening and closing between the different microbubble sizes become less significant [121]. At the same time, in the single-factor investigation of microbubble concentration, it was found that higher dose could cause stronger damage to BBB [122]. Another interesting research found that the BBB opening efficiency increased 10-fold with the diameter of microbubbles from 6 *μ*m to 2 *μ*m at a fixed concentration [123]. However, when the size and concentration of microbubbles were merged into the volume dose, there was no significant difference in the half-life for in vivo ultrasound contrast persistence in mice with the same volume dose. Therefore, the volume dose of microbubble may be a new direction to investigate the therapeutic effect of drug-loaded microbubbles. By increasing the concentration of microbubbles prepared, it is expected to improve the therapeutic efficiency of drug-loaded microbubble [124, 125]. As the important characterization parameter of drug-loaded microbubbles, entrapment efficiency refers to the ratio of the amount of drugs wrapped into microbubbles to the total dosage and drug-loading capacity refers to the ratio of the amount of medicine wrapped into microbubbles to the total weight of the microbubble [126]. Drug-entrapment efficiency and drug-loading capacity directly affect the drug release concentration of drug-loaded microbubbles in the target site, so how to improve the above two parameters is the key to improve the therapeutic efficiency [127]. Lipids and proteins have been widely used in the preparation of drug-loaded microbubbles because of their good biocompatibility, but the poor stability in vivo is an important factor restricting their development. PEGylation can effectively improve the stability of microbubble in vivo. Upadhyay et al. found that the stability of PEGylated BSA and DSPC-PEG40S microbubbles was significantly higher than that of non-PEGylated BSA microbubbles and showed almost no immunogenicity in immunogenic studies [128]. To further improve the stability of drug-loaded microbubbles, degradable polymers with higher mechanical strength began to be used in the preparation of microbubbles like poly (lactic-co-glycolic acid) (PLGA) and PLA. The PTX-loaded microbubbles prepared by PLGA showed obvious slow release efficiency, and the ultrasound imaging time in vivo lasted longer than that of the SonoVue microbubbles [129]. In addition to the influence of the physical and chemical properties of drug-loaded microbubbles on the therapeutic efficiency, the ultrasonic-related parameters used to stimulate microbubbles will also be the factors affect the therapeutic efficiency, like ultrasonic intensity, mechanical index, and duty cycle, but the correlation between microbubbles and therapeutic efficiency needs to be further studied systematically [120].

However, there are still many problems that need to be solved in clinical applications. (1) Since the drugs are adhered on the surface of the microbubbles or incorporated into the phospholipid layer and the microbubble shell is thin, drug loading is limited. Although the amount of loaded drugs can be increased by developing the drug liposomes on the surface of the microbubbles, it is still necessary to use other emerging technologies to increase the drug-loading capacity. (2) The stability of microbubbles is poor and its circulation time is short. After intravenous injection, only a small number of drugs can reach the tumor sites through the circulation. Much more efforts should be made to achieve a long circulation time for delivering drugs to target sites because the local drug concentration will increase after several cycles. It is quite necessary to optimize the formulation to prolong the storage stability and in vivo stability for clinical applications. It was found that the use of low-intensity ultrasound could increase the adhesion of microbubbles in the blood vessel wall without destroying the microbubbles, thereby prolonging the cycle time of the microbubbles. (3) The safety and effectiveness should be further investigated when drug-loaded microbubbles are used in the treatment of cancer and thrombus. The ultrasound parameters and time should also be optimized to obtain better treatment. Although there are still some problems in the applications of microbubbles combined with ultrasound, this technology provides a novel approach for the treatment of thrombus and tumor. Believing that with further exploration in drug-loaded microbubbles, it can play a greater role in the treatment of clinical diseases.

## Figures and Tables

**Table 1:** The combination of drug-loaded microbubbles and ultrasound for tumor treatment.

Drug	Type of microbubble	Microbubble size	Type of tumor	Application	Ref.
Doxorubicin	Lipid MB	4.00 *μ*m	Malignant glioma	Exploring the inhibition ratio to human glioblastoma cells	[68]
Carmustine	Lipid MB	1.32 *μ*m	Glioblastoma multiforme	Investigating the treatment efficacy in rat glioma model	[69]
Doxorubicin	Lipid MB	1.04 *μ*m	Glioblastoma multiforme	Investigating the efficiency of opening BBB and drug delivery	[70]
Docetaxel	Lipid MB	623.10 nm	Liver tumor	Inhibiting tumor growth in a rabbit liver tumor model	[30]
Hydroxycamptothecin	Lipid MB	1.48 *μ*m	Liver tumor	Increasing the rate of tumor inhibition	[71]
Doxorubicin	Poly(lactic acid) MB	1.50 *μ*m	Liver tumor	Achieving the treatment in rabbit liver tumor model	[72]
Doxorubicin	Lipid MB	1.02 *μ*m	Pancreas carcinoma	Achieving the treatment of pancreatic cancer in rat model	[31]
Docetaxel	Lipid MB	3.30 *μ*m	Colon adenocarcinoma	Investigating the antitumor effect on human colon adenocarcinoma cell line	[73]
Paclitaxel	Lipid MB	1.68 *μ*m	Breast cancer	Achieving the treatment in mice breast cancer model	[74]
Doxorubicin	Lipid MB	1.64 *μ*m	Breast cancer	Investigating the antitumor effect on human breast cancer cells	[75]
Paclitaxel	Lipid MB	1.80 *μ*m	Ovarian cancer	Investigating the antitumor effect on human ovarian carcinoma cells	[76]
Paclitaxel	Lipid MB	1.80 *μ*m	Ovarian cancer	Achieving the treatment of in mice ovarian cancer model	[77]
